# Mid-infrared photoacoustic gas monitoring driven by a gas-filled hollow-core fiber laser

**DOI:** 10.1038/s41598-021-83041-2

**Published:** 2021-02-10

**Authors:** Yazhou Wang, Yuyang Feng, Abubakar I. Adamu, Manoj K. Dasa, J. E. Antonio-Lopez, Rodrigo Amezcua-Correa, Christos Markos

**Affiliations:** 1 DTU Fotonik, Department of Photonics Engineering, Technical University of Denmark, 2800 Kgs. Lyngby, Denmark; 2 COPAC A/S, Diplomvej 381, 2800 Kongens Lyngby, Denmark; 3 CREOL, The College of Optics and Photonics, University of Central Florida, Orlando, FL 32816 USA; 4 NORBLIS IVS, Virumgade 35D, 2830 Virum, Denmark

**Keywords:** Fibre lasers, Mid-infrared photonics, Optical sensors

## Abstract

Development of novel mid-infrared (MIR) lasers could ultimately boost emerging detection technologies towards innovative spectroscopic and imaging solutions. Photoacoustic (PA) modality has been heralded for years as one of the most powerful detection tools enabling high signal-to-noise ratio analysis. Here, we demonstrate a novel, compact and sensitive MIR-PA system for carbon dioxide (CO_2_) monitoring at its strongest absorption band by combining a gas-filled fiber laser and PA technology. Specifically, the PA signals were excited by a custom-made hydrogen (H_2_) based MIR Raman fiber laser source with a pulse energy of ⁓ 18 μJ, quantum efficiency of ⁓ 80% and peak power of ⁓ 3.9 kW. A CO_2_ detection limit of 605 ppbv was attained from the Allan deviation. This work constitutes an alternative method for advanced high-sensitivity gas detection.

## Introduction

Direct access to the MIR spanning from 3 to 20 µm, is of tremendous scientific and industrial interest as it covers the main absorption lines of molecules of great importance, such as alkanes or greenhouse gasses^1,2^. Thereby it can be used to unambiguously detect the molecular composition of a broad variety of gases, liquids, and even solids in a non-intrusive way^3^. An important greenhouse gas with a crucial role in environmental monitoring^4^, agricultural production^5^, food quality testing^6^ and combustion diagnostic^7^, is CO_2_. Although there are several well-established approaches on CO_2_ detection, optical spectroscopy remains still perhaps the most effective one owing to its unique advantages of high sensitivity, fast response time, long lifetime, and long-term stability^8,9^. There are several optical methods on gas detection^10–16^, and some of them have already been well-developed to implement CO_2_ monitoring outdoor and in harsh conditions^10–14^. The dominant absorption band of CO_2_, located at ~ 4.3 μm wavelength, is 10 times higher than its second strongest absorption peak at 15 μm and two orders of magnitude higher than its third strongest absorption peak at 2.7 μm, indicating that MIR band enables the ability to achieve high sensitivity and concentration range compared to its counterpart in the near-IR.

On the other hand, PA technology constitutes the cornerstone for more than 20 years towards high-resolution gas spectroscopy and imaging^17–20^. Its underlying mechanism relies on the photo-excited acoustic signals that linearly vary as a function of the absorbed laser power. This emerging tool has been presently established within industrial gas analysis sector because compared with other optical detection schemes, it possesses unique advantages such as high-sensitivity, wide-dynamic range, short optical path length (compact size), and near-to-zero background signal^21–27^. The acoustic signals can be excited either from a modulated (square or a sine wave obtained by modulating CW laser with a Hz or kHz repetition rate) or pulsed (nanosecond pulse duration) laser pump. The PA amplitude from the acoustic resonator is proportional to the average power of the CW laser in the former, while to the pulse energy in the latter case^22^. Therefore, high average power (CW) or pulse energy is an essential factor for generation of enhanced acoustic signals, to enable high signal-to-noise ratio and wide concentration range of the target gas. Quantum and interband cascade laser (QCL and ICL) technology, is well-known for gas detection because they offer narrow linewidth, good stability, as well as wide wavelength tunability by adding an extra grating to form an external cavity structure^28^. However, QCLs as well as ICLs have low peak power and pulse energy, thus limiting their use in pulse-based PA gas spectroscopy^29–31^. The ability to combine active Raman gases—as the nonlinear medium—with the recently developed low-loss anti-resonant hollow-core fibers (ARHCF) platform allowed the scientific community to move gas-based nonlinear optics to remarkable and previously inaccessible parameter regimes of high intensity with MHz level bandwidths^32^, and therefore create a desirable solution for the generation of high peak power and thus high-energy laser pulse in the UV and MIR, as demonstrated by several impressive results recently^33–39^. Despite these advantages, as an emerging research field, truly viable applications have not yet been explored, to the best of our knowledge.

Here, we report for the first time the development of a novel “hybrid” technology by integrating a H_2_-based fiber Raman laser as the excitation source with a compact PA system for CO_2_ monitoring at 4.22 μm, in the MIR. The source is based on vibrational stimulated Raman scattering (SRS) in a hydrogen (H_2_)-filled ARHCF pumped by a compact custom-made 1532.8 nm fiber laser with 6.9 ns long pulses and ~ 11 kW peak power. The output laser has an energy of 17.6 μJ and peak power of ~ 3.9 kW, enabling a CO_2_ detection as low as ~ 600 ppbv (see state-of-the-art in Supplementary Table S1). Moreover, the MIR PA scheme presented in this paper, offers a flexible selection of laser parameters with respect to the targeted trace gas detection application and promises a new tool for PA imaging and spectroscopy.

## Results

### Theory

**Figure 1:**
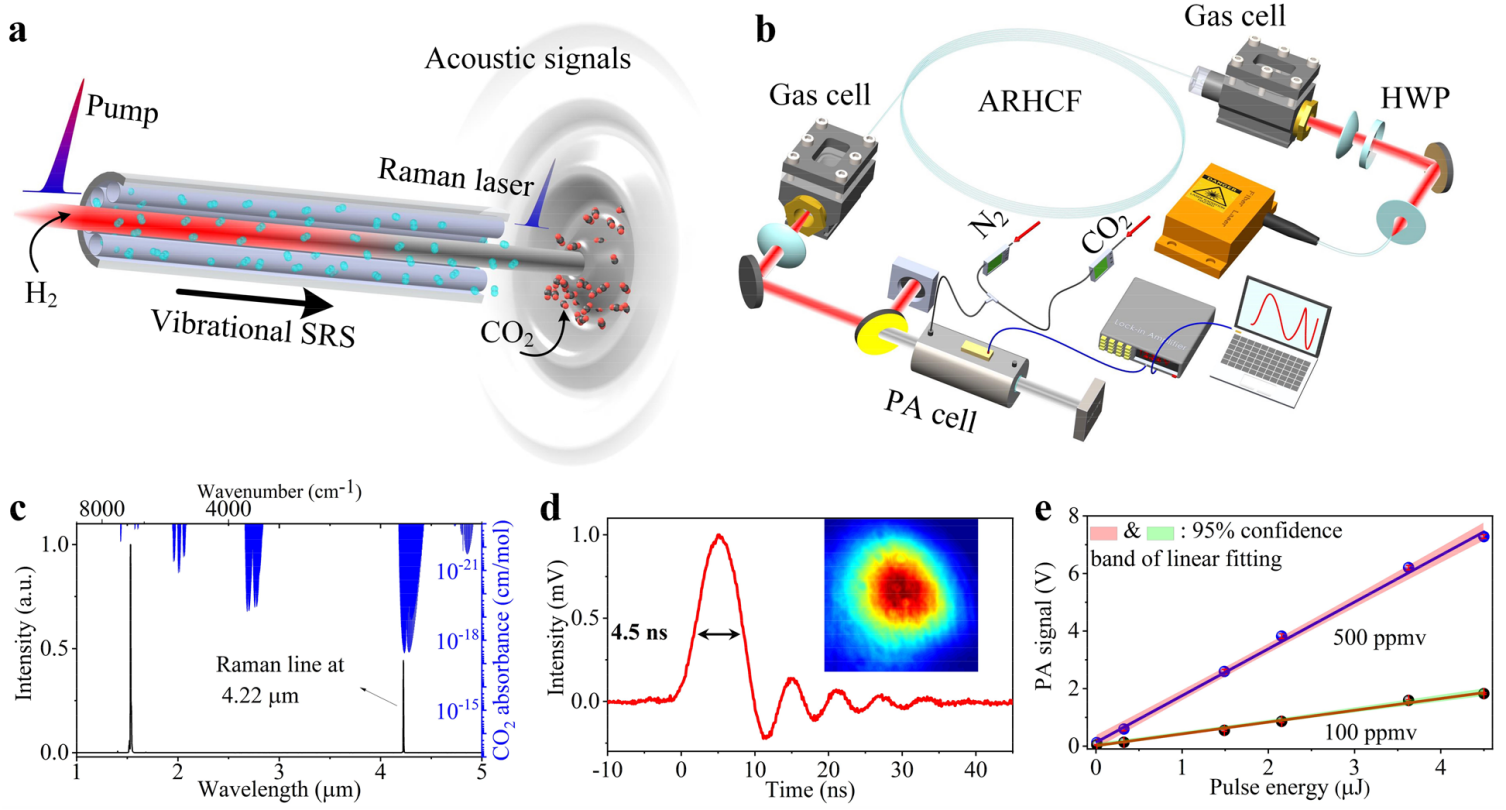
Principle and schematic of the MIR PA CO_2_ monitoring. (**a**) Concept of the PA system integrated with an H_2_-filled ARHCF based MIR Raman laser. (**b**) Experimental setup, primarily consisting of a pump laser, H_2_-filled ARHCF, PA cell, a power meter to record the optical power of the laser, a LIA and a dynamic N_2_/CO_2_ gas-flow configuration. (**c**) The spectrum of the pump and generated laser overlaid on top of the simulated absorbance spectrum of CO_2_. The right axis shows the simulated absorbance spectrum of CO_2_. (**d**) Pulse profile of the Raman laser; Inset is the measured beam profile in the MIR. (**e**) Measured PA intensity as a function of the laser pulse energy at two different CO_2_ concentrations (500 ppmv and 100 ppmv). Shaded regions correspond to the 95% confidence band for every case.

The concept of the proposed gas-based excitation PA system is shown in Fig. 1a. The MIR pulses are generated through the vibrational SRS by using H_2_ as nonlinear propagation medium, which is pressurized in the hollow region of ARHCF and can directly red-shift the near-infrared pump line to mid-infrared region due to its large Raman shift coefficient of 4155 cm^−1^. Meanwhile, the use of ARHCF is able to confine most energies of the pump beam as well as the generated Stokes laser beam within the fiber core region with a diameter of 71 μm here, therefore ensuring intense H_2_-light interaction. Consequently, the output MIR laser overlapping with the strongest CO_2_ absorption line of ~ 4.3 μm, can be directly and efficiently converted from pump pulses at ~ 1533 nm wavelength, which is still within the gain band of the Er^3+^-doped fiber laser technology. The MIR output pulses then propagate through the CO_2_-filled PA cell, leading to a localized transient heating and expansion of the gas molecules, followed by a pressure (PA) wave which travels radially outwards from the interaction region^40,41^. The intensity of the PA wave depends on the absorption coefficient and thus, according to the Beer–Lambert law, on the concentration of CO_2_^21^. In practice, the excited PA wave usually has a relatively weak intensity and thus a geometrically-optimized PA cell is necessary to enhance the generated PA intensity through constructive acoustic resonances. Given a pulsed excitation source, the PA amplitude can be expressed as^22^

1 $$P = \frac{(\gamma - 1)C}{V}\alpha E,$$

where *P* is the PA amplitude, *C* is a factor that depends on the position of the laser beam and the transducer relative to the selected eigenmode distribution, *γ* is the adiabatic coefficient of the gas, *V* is the volume of the PA cell, *α* is the optical absorption coefficient of the gas at the excitation wavelength, and *E* is the laser pulse energy. According to Eq. (1), the pulse energy plays a key role in the PA’s intensity and thus the sensitivity of the system. In our design, we optimized the pulse energy of the excitation laser by selecting an appropriately long pump pulse duration that allows the efficient suppression of the transient Raman regime (see analysis in Supplementary Note 1 and 2).

### Experiment setup

The experimental setup, shown in Fig. 1b, consists of the H_2_-filled fiber Raman laser source and the PA detection forming the final system. The excitation source is based on an H_2_-filled ARHCF, sealed by two custom-made high-pressure gas cells with a size of 5 cm × 4 cm × 5 cm. The ARHCF was designed and numerically optimized using a finite element method (see Supplementary Note 3), in order to obtain the two main anti-resonant guiding windows at the pump (i.e. 1532.8 nm) and the generated wavelength (i.e. 4.22 μm). The fiber was fabricated using the stack-and-draw method (see “Methods”). The pump is a custom-made linearly polarized fiber laser delivering a pulse train at a repetition rate of 8 kHz with a duration of 6.9 ns (see “Methods”). The pulse energy of the pump is calculated to be ~ 80 µJ, corresponding to an estimated peak power of ~ 11 kW. The center wavelength and linewidth measured by an optical spectrum analyzer (ANDO AQ6317B, AssetRelay) with a resolution of 0.01 nm, are 1532.8 nm and ~ 0.06 nm, respectively. A half-wave plate (HWP) is placed before the ARHCF to optimize the efficiency of the SRS process^42^. The output beam from the second gas cell is collimated using a CaF_2_ lens with a focal length of 3 cm. A 2.4 µm long pass filter with 80% transmission at 4.2 µm is used to extract the Raman beam from the residual pump.

In the PA detection arm, the excitation laser passes through a PA cell (outer dimensions of ~ 10 cm length and 4 cm diameter) filled with CO_2_ at a specified concentration. The concentration of the CO_2_ is controlled with an accurate dynamic gas distribution method that involves dilution of the initial 500 ppmv CO_2_ concentration with pure N_2_ using two mass flow controllers (MFCs) (see “Methods”). The PA signals detected by the microphone are amplified using a low noise band-pass amplifier and a lock-in amplifier (LIA). The output beam from the other side of the PA cell is simultaneously monitored using a thermal power detector.

### Characterization of H_2_-filled ARHCF laser and PA signal

The maximum MIR emission was obtained at the maximum pump power and an optimal fiber length of 3.95 m. When the ARHCF length is longer than the optimal one, the generated power starts to decrease due to the increased fiber loss at the pump as well as the MIR signal wavelength. In the optimal case, the Raman laser is generated when the H_2_ pressure is higher than 5 bar. Then, the power steadily scales up to saturation (maximum) value of ~ 141 mW at a pressure of 20 bar (see Supplementary Note 4). The maximum pulse energy is up to 17.6 µJ, corresponding to a 74% quantum efficiency excluding the absorption losses from the bulk optics in the experimental setup (see Supplementary Note 4). Both the pulse energy and quantum efficiency are the highest reported in gas-filled fiber lasers beyond 3 µm wavelength^35,37,39,43–45^. Furthermore, the H_2_ pressure of 20 bar required to achieve the highest pulse energy is significantly less than other reported results^37,43^, since in this work the transient Raman regime is efficiently suppressed by using long duration (6.9 ns) pump pulses.

Figure 1c shows the measured spectrum of the Raman laser from near-IR to MIR using an optical spectrometer (Spectro 320, Instrument Systems), as well as the absorbance spectrum of CO_2_ adopted from the high-resolution transmission molecular absorption database (HITRAN)^46^. It can be seen that the generated laser line at 4.22 µm overlaps with the strongest absorption band of CO_2_ as initially designed. The linewidth is expected to be in the range of hundreds of MHz to a few GHz, which cannot be resolved by the spectrum meter with a minimum resolution of 0.3 nm. The generated pulse profile at 4.22 µm is measured using a 100 MHz MIR photodetector (PDAVJ10, Thorlabs) as shown in Fig. 1d. The measured pulse duration of ~ 4.5 ns is less than the pump duration of ~ 6.9 ns due to the threshold of SRS. The peak power is thus calculated to be ~ 3.9 kW using a Gaussian pulse approximation. The oscillation in the trailing edge is caused by the limited bandwidth of the MIR photodetector. For the same reason, the actual pulse duration is slightly less than the measured value of ~ 4.5 ns. The inset of Fig. 1d corresponds to the beam profile of the Raman laser measured by an uncooled terahertz imager (IRV-T0831, NEC), indicating the Gaussian-like power distribution of the fundamental mode in the ARHCF.

The PA wave was detected when the collimated MIR beam interacts with the CO_2_ molecules inside the PA cell. The recorded waveform of the PA signal generated from a constant CO_2_ concentration of 500 ppmv has a sudden onset of an acoustic oscillation followed by a slow decay^47^. The amplitude of the first oscillation is proportional to the released energy of the corresponding heat pulse and thus the laser pulse energy^47^. This observation is supported by Fig. 1e, showing how the PA intensity obtained from the LIA linearly scales with the pulse energy. The slope of the linear regression is related to the CO_2_ concentration. The pulse energy in this plot only reaches 4.5 µJ (corresponding to 36 mW average power), which is less than the maximum pulse energy of 17.6 µJ mentioned above. This is mainly attributed to the losses from the long-pass filter with 80% transmission at 4.2 µm, combined with the optical absorption of CO_2_ in ambient air. The latter leads to a loss of up to ~ 58% due to the ~ 1 m distance between the output gas cell and the PA cell (see Supplementary Note 5).

### CO_2_ detection and analysis

**Figure 2:**
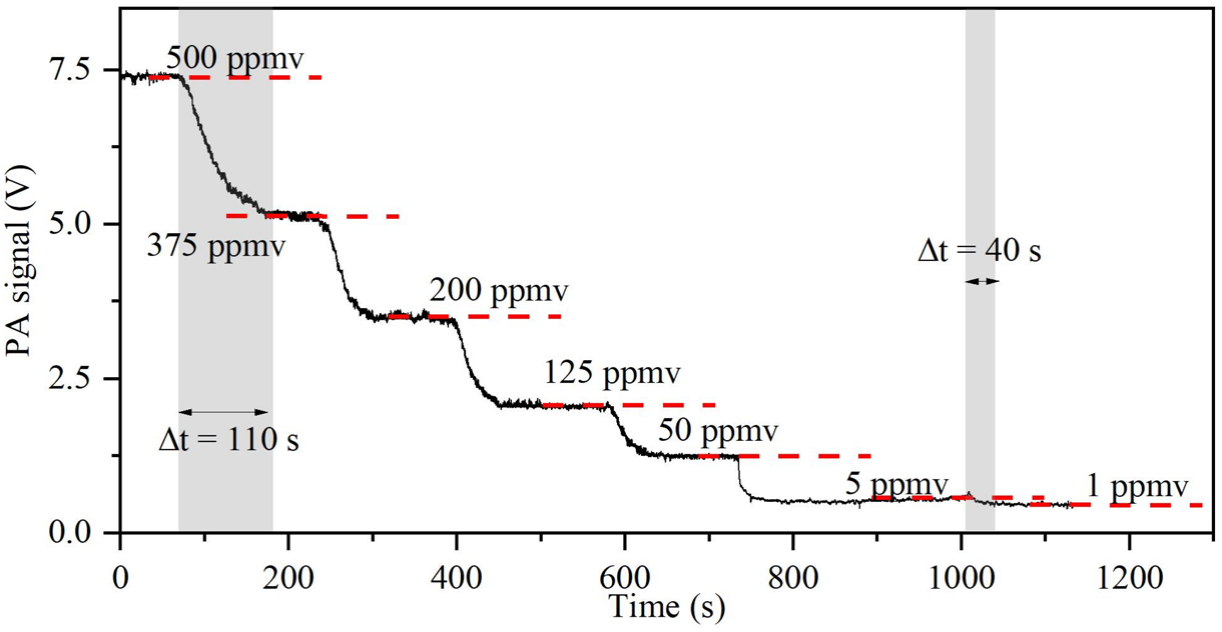
PA intensity evolution as a function of time when CO_2_ concentration decreases from 500 to 1 ppmv.

Figure 2 shows the dynamic evolution process of the measured PA intensity with respect to different CO_2_ concentrations over a range of 500 ppmv–1 ppmv. It should be emphasized that low gas concentrations cannot be simply determined here by monitoring the absorption through the optical power. In order to support this claim, the optical power was recorded in parallel with the PA signal, indicating the random power changes with varying concentrations (see Supplementary Note 6). During the PA monitoring, the PA cell was under a constant flow of CO_2_ at a specific concentration. The equilibrium between two consecutive concentrations is ranged from ~ 40 s (minimum) to ~ 110 s (maximum), including also the manual adjustment time of the MCFs for every concentration (~ 10 s), as well as the gas diffusion time from the MCFs to the PA cell, connected by a 1.5 m gas tube (6 mm inner diameter). The 4 mm inner diameter of the PA cell is comparable with the gas tube, but it has a short length of only 10 cm, indicating that the gas diffusion time inside the PA cell is far shorter than that of the gas tube.

**Figure 3:**
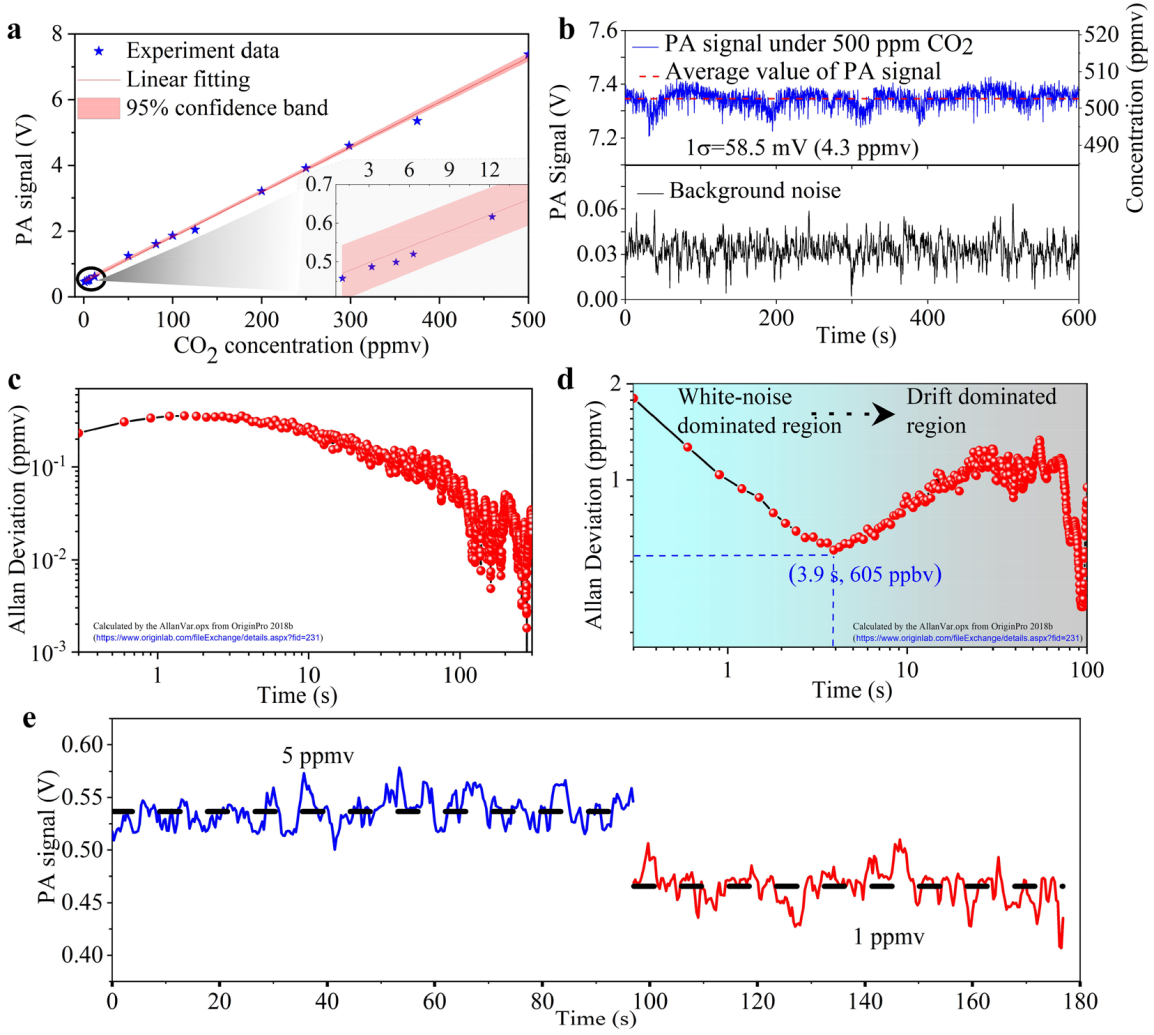
CO_2_ monitoring results. (**a**) PA intensities measured at different CO_2_ concentrations ranging from 0.5 ppmv to 500 ppmv. Inset: zoom of (**a**) with the range of 0.5 ppmv to 15 ppmv. (**b**) Top: stability measurement of the PA signal at a constant CO_2_ concentration of 500 ppmv, with 3 sccm gas flow; Bottom: background noise by turning off the laser (the gas flow keeps flushed at 3 sccm). (**c**) and (**d**) are calculated Allan deviations corresponding to bottom and top curves of (**b**). (**e**) PA signals obtained at 5 ppmv and 1 ppmv CO_2_ concentrations.

Figure 3a shows the measured PA signal at different CO_2_ concentrations, revealing the linear dependence of the PA intensity on the concentration. The linear function extracted from the slope of Fig. 3a, can be expressed as *I* = 0.0137 × *C* + 0.46, where *I* and *C* denote PA intensity (unit: V) and CO_2_ concentration (unit: ppmv), respectively. This linear regression acts as a calibration model to calculate the CO_2_ concentration from the corresponding PA intensity. The top sub-figure in Fig. 3b shows the stability measurement of the PA signal at a constant concentration of 500 ppmv CO_2_ over 600 s under a continuous flow of CO_2_ into the PA cell. The *1σ* standard derivation was found to be 32.0 mV, corresponding to a concentration of 2.33 ppmv. The measured signal is accompanied by drift over the 600 s monitoring that is induced by the power drift of the Raman laser (see Supplementary Note 7). The background noise recorded by turning off the laser is also presented for comparison (see bottom sub-figure in Fig. 3b). It can be seen that the background noise fluctuates without obvious drift, indicating that the signal drift is dominated by the laser. The calculated 1σ standard derivation is 7.6 mV, corresponding to 557 ppbv. The Allan deviation, which is widely used for the evaluation of a system’s performance in terms of the detection limit as a function of integration time^48^, is shown in Fig. 3c,d based on the stability measurements of Fig. 3b. In Fig. 3c, it can be seen that the Allan deviation initially exhibits a weak increase as the integration time increases, this is then followed by an approximate linear drop in the logarithmic scale. This decrease is a sign that the background noise mainly obeys a white noise distribution and the drift is negligible^48^. In Fig. 3d, for an initial integration time of 300 ms, the detection limit is 1.45 ppmv, which is consistent with our experimental observations in Fig. 3e, showing a direct comparison of PA intensities at two different CO_2_ concentrations. The calculated 1*σ* standard deviation of 1.2 ppmv at a fixed CO_2_ concentration of 5 ppmv concentration and 1.06 ppmv at 1 ppmv are in agreement to the Allan deviation of 1.45 ppmv (at 500 ppmv CO_2_ concentration). When the integration time increases, the system first enters a white-noise dominated region before reaching an optimum integration time. After this optimum value, the system enters the drift-dominated region, which includes various intrinsic and extrinsic “drift” noises such as power instability, temperature fluctuations, etc.^48^. The detection limit of our system was found to be ~ 600 ppbv at the optimal integration time of 3.9 s. The optimal integration time is relatively short when compared to other similar reports^49–51^, and the reason is due to the power instabilities of the Raman laser originating from the random quantum noise during the initialization of the vibrational SRS (see Supplementary Note 7). For the detection limit of ~ 605 ppbv at an integration time of 3.9 s, the CO_2_ absorption coefficient is estimated to be ~ 0.13 cm^−1^. The lock-in amplifier bandwidth is 0.032 Hz with a filter roll-off rate of 12 dB/octave. Therefore, the normalized noise equivalent absorption is calculated to be ~ 0.0262 cm^−1^ W/√Hz.

## Discussion

Pertaining to the unique features of the PA-based gas detection approach, it is shown that the use of a MIR high pulse energy and peak power H_2_-filled Raman fiber laser acts as a new element that significantly scales up the performance of a PA system and creates new research directions towards gas spectroscopy and microscopy^19^. With respect to this regard, our investigation was focused on CO_2_ monitoring and demonstrated a detection of ~ 600 ppbv at 3.9 s integration time. Moreover, the detection limit of the proposed system can be further improved by suppressing the noise of the Raman laser, that is built-up from the random quantum noise^52,53^. In order to support our claim, we measured a pulse train of both the initial pump and also the generated MIR laser comparing and indicating the randomly distributed pulse-to-pulse fluctuations (see Supplementary Note 7). The relative standard deviation of pulse energy over 5000 consecutive pulses, was measured to be ~ 3% for the pump while up to ~ 17% for the generated Raman laser which inevitably compromises the signal-to-noise ratio of the PA signal. This is also reflected by monitoring the average power of the pump as well as the output MIR beam from the PA cell (see Supplementary Fig. S6). The wavelength modulation method is a possible way to alleviate the laser power noise, but it needs the laser to operate in CW state. This is however challenging for gas-based Raman laser generation, because the occurrence of stimulated Raman scattering requires high laser intensities (corresponding to several kW peak power for the ARHCF with a core diameter of tens of micrometers). A more feasible solution to tackle the relatively high intensity fluctuation could be the use of an additional low-noise light source within the Raman gain band, in order to suppress the influence of the quantum noise.

In summary, CO_2_ monitoring with high sensitivity is demonstrated by combining the powerful PA-based detection scheme with the emerging MIR gas-filled fiber laser technology. The excitation source relies upon an H_2_-filled ARHCF technology to achieve a direct and efficient SRS conversion from a narrow linewidth fiber laser pump delivering 7 ns pulse with 11 kW peak power at 1532.8 nm. The quantum efficiency of the generated MIR Raman laser at 4.22 μm (strongest CO_2_ absorption band) is about 74%, with a high pulse energy of 17.6 µJ and peak power of ~ 3.9 kW. The development of such excitation parameters enabled the generation of intense PA signals from the CO_2_ molecules, achieving a predicted detection limit of ~ 600 ppbv.

## Methods

### Design and fabrication of ARHCF

The designed ARHCF is produced by stacking high-purity silica capillaries inside a larger silica tube, to make the bulk macroscopic replica of the intended fiber, known as the *preform*. The preform is then fed into a fiber drawing tower, where the glass is heated up to 1650 °C, just above the glass transition temperature of fused silica. The softened glass is then vertically drawn at a controlled speed, to attain the desired fiber dimensions. Unlike conventional solid-core fibers, the presence of air hollow regions in the preform adds a level of complexity in the drawing process since the risk of hole-collapsing is high. To mitigate this challenge, during fiber drawing we used distinct gas pressure lines into each hollow region in the preform in order to maintain a uniform geometric structure as the preform passes through the hot-zone in the furnace.

### Pump fiber laser

**Figure 4:**
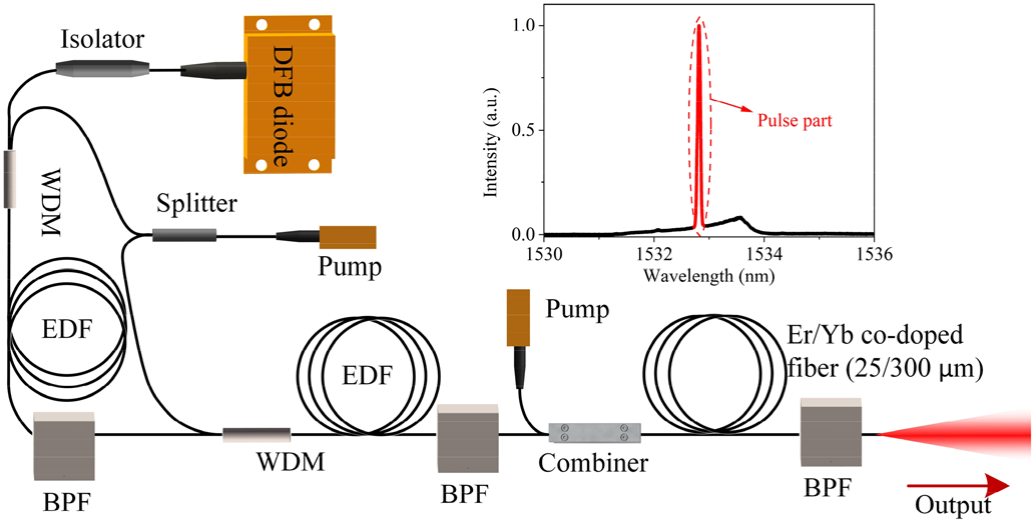
Configuration of the custom-made 1.53 μm pump fiber laser. Inset: measured spectrum of the output laser.

The pump laser is built using a directly modulated distributed feedback laser (DFB) diode-based all-polarization maintaining master oscillator power amplifier configuration, as shown in Fig. 4. The DFB diode laser is modulated through a pulse generator to deliver linear polarized 7 ns pulses at a repetition rate of 8 kHz. The seed laser is initially amplified using two stages of Er-doped fiber pre-amplifiers core-pumped by sharing the same CW pump laser diode through a laser splitter, and then amplified by a double-clad co-doped Erbium: Ytterbium fiber power amplifier cladding-pumped by 915 nm CW laser diode with 10 W maximum output power. An important limiting factor for the amplification efficiency of the seed laser is the reabsorption effect of Er^3+^ ions from ground-state of ^4^I_15/2_ to upper-level of ^4^I_13/2_, which significantly transfers the 1533 nm laser to amplified spontaneous emission (ASE) at a longer wavelength, and thus limits the net gain around that wavelength. Therefore, in order to suppress the reabsorption effect, three bandpass filters (BPFs) with ~ 2 nm bandwidth are used after each amplifier.

The maximum output power of the fiber laser is 1.4 W. By excluding the contribution of ASE, the maximum average power of the pure pulse signal is estimated to be ~ 640 mW. With 8 kHz repetition rate, the pulse energy and peak power are calculated to be ~ 80 µJ and ~ 11 kW, respectively. The measured polarization extinction ratio (PER) of the total output is − 19 dB. Because the ASE part is unpolarized, the PER of the pulse signal is actually higher than this value.

### PA signal acquirement and analysis system

The PA cell adopts the conventional cylindrical structure, i.e., a single hollow-core cylinder region running through a stainless steel housing. The cylinder with a small radius of ~ 4 mm and a short length of ~ 10 cm places its acoustic eigenmodes within the broad spectrum range of the PA signals excited by the pulsed laser^54^. Such a compact dimension of the cell significantly shortens the gas diffusion time and enables fast monitoring of the target gas. The inner surface of the cylinder is well-polished to ensure the uniformity of the acoustic reflections. Sapphire glass serves as the optical windows at both ends of the cylinder where the beam enters/exits. Buffer regions are designed to avoid the acoustic excitation disturbances caused by the absorption of optical windows and the environmental acoustic noise. An acoustic microphone (KECG2740, Kingstate Electronics Corp.) which is the crucial component of the PA cell was placed halfway between the ends of the cylinder. It has a frequency range of 20 Hz–20 kHz. The sensitivity and signal to noise ratio are 10 mV/Pa and 56 dBA at 1 kHz frequency. The microphone diameter is 6 mm. The signal from the microphone is first amplified by a low-noise custom-made preamplifier and is then sent to an LIA (SR530), which is synchronized to the pulse generator of the pump fiber laser. During the experiment, the sensitivity of the LIA was appropriately chosen, to acquire accurate PA data without signal saturation.

### CO_2_ concentration control system

To control the concentration of CO_2_ in the PA cell, high purity N_2_ is used as a diluent gas to change the concentration from 500 to 1 ppmv. N_2_ is a homonuclear diatomic molecule, and thus, does not absorb IR radiation and is a suitable mixing gas for MIR spectroscopy. In the experimental setup, both CO_2_ and N_2_ gas bottles are connected to individual MFCs (El-Flow, Bronkhorst High-Tech), where the flow-rates are regulated for a pre-determined concentration. The mixed gas is then flushed through the PA gas cell and the real-time concentration can be read through the PA intensity after the LIA. For the Allan deviation measurement, the flow was set to 3 sccm. This is an attempt to eliminate any additional noise that might arise from the high turbulent flow of gas into the chamber during the sensitive measurement. Since pressure inside the gas-cell is maintained at 1 atm throughout the experiment, there is no increase in concentration as a result of pressure build-up in the gas-cell. This is attained by leaving the exit orifice on the gas-cell open, whenever gas is purged.

## Supplementary Information

Supplementary Information.

## Data Availability

The data that support the findings of this study are available from the corresponding author upon reasonable request.
