# Supplementary material for: Mid-infrared photoacoustic gas monitoring driven by a gas-filled hollow-core fiber laser
Source: Sci Rep. 2021 Feb 10;11:3512. doi: 10.1038/s41598-021-83041-2 (PMC7876039; doi:10.1038/s41598-021-83041-2)
Supplement: Supplementary file 1 — Supplementary Information. [file 41598_2021_83041_MOESM1_ESM.docx]

Mid-infrared photoacoustic gas monitoring driven by a gas-filled hollow-core fiber laser

Yazhou Wang,^1*^ Yuyang Feng, ^2^ Abubakar I. Adamu, ^1^ Manoj K. Dasa, ^1^ J. E. Antonio-Lopez, ^3^ Rodrigo Amezcua-Correa,^3^ and Christos Markos^1,4^

^1^ DTU Fotonik, Department of Photonics Engineering, Technical University of Denmark, 2800 Kgs. Lyngby, Denmark

^2^ COPAC A/S, Diplomvej 381, 2800 Kongens Lyngby, Denmark

^3^ CREOL, The College of Optics and Photonics, University of Central Florida, Orlando, Florida 32816, USA

^4^ NORBLIS IVS, Virumgade 35D, 2830 Virum, Denmark

*Correspondence to [yazwang@fotonik.dtu.dk](mailto:yazwang@fotonik.dtu.dk)

**Supplementary Table S1.** Summary of state-of-the-art of detection limit on CO_2_ monitoring using optical methods.

| Detection limit | Method | Excitation source | Background gas | Year | Reference |
| --- | --- | --- | --- | --- | --- |
| 1 ppm | Non-dispersive infrared | Microbulb ( Broadband MIR light source) | N_2_ & O_2_ | 2013 | 1 |
| 230 ppm | Photoacoustic | QCL (4.3 μm) | Biogas | 2006 | 2 |
| <5 ppm | Cross-band, two-line thermometry | QCL(~ 4.2 μm) & diode laser (~2.7 μm) | N/A | 2014 | 3 |
| 30 ppm | Non-dispersive infrared | Heat source (Broadband MIR light source) | N_2_ | 2016 | 4 |
| 30 ppm | Non-dispersive infrared | micro-hotplate IR-source (~4.26 μm) | Dry air | 2013 | 5 |
| 4.54 ppmv | Time division multiplexing technique | A unclear broadband MIR light source | N_2_ | 2017 | 6 |
| 30 ppb | Photoacoustic | Diode laser (~ 2.7 μm) | N_2_ | 2008 | 7 |
| 5 ppq  (^14^C^16^O_2_) | Saturated-absorption cavity ring-down spectroscopic technique | QCL(~ 4.2 μm) | ^12^C^16^O_2_ | 2016 | 8 |
| 300 ppb | Photoacoustic | Diode laser (~ 1.57 μm) | Ar | 2007 | 9 |
| 300 ppt | Photoacoustic | Quantum cascade laser (~ 4.33 μm) | N_2_ | 2014 | 10 |
| 18 ppm | Photoacoustic | Diode laser (2 μm) | N_2_ humidified by H_2_O vapor | 2007 | 11 |
| **600 ppbv** | **Photoacoustic** | **Gas-filled Raman fiber laser (4.22 μm)** | **N_2_** | **2021** | **This work** |

**Supplementary Note 1: Raman laser model**

The Raman laser was modeled based on the following equations^12^:

$\begin{matrix} \frac{\partial E_{s}}{\partial z}=-i\gamma_{1}Q^{*}E_{p}-\alpha_{s}E_{s} \\ \frac{\partial E_{p}}{\partial z}=-i\frac{\lambda_{s}}{\lambda_{p}}\gamma_{1}QE_{s}-\alpha_{p}E_{p} \\ \frac{\partial Q}{\partial t}+\frac{Q}{T_{2}}=-i\gamma_{2}E_{p}E_{s}^{*} \end{matrix}$ (1)

where *E_p_* and *E_s_* are the complex amplitudes of the pump and Stokes waves, respectively. They are coupled through the quantity of macroscopic polarization *Q*. *z* is the propagation distance along the fiber, and *T* is the time in the retarded frame. *v_s_* and *v_p_* are the group velocities of the pump and Stokes lasers, respectively, and similarly, α*_p_* and α*_s_* are the corresponding optical losses. 𝜆_p_ and 𝜆_s_ are the wavelengths of the pump and Stokes waves. *T*_2_ is the dephasing time of the molecular vibration (H_2_ in our case). γ_1_ and γ_2_ are coupling constants, and they are expressed as follows:

$\gamma_{2}=\frac{1}{2}\sqrt{\frac{\text{g}_{R}}{A_{eff}CT_{2}}}$ (2)

$\gamma_{1}=C\gamma_{2}$ (3)

$\text{C}=\frac{4\pi\omega_{s}^{2}(\omega_{p}-\omega_{s})\lambda_{s}}{c^{2}}$ (4)

where *A_eff_* is the effective mode area of the ARHCF, *g_R_* is the gain coefficient of the H_2_ at the steady Raman regime and is pressure-dependent as^13^:

$\text{g}_{R}=9.37\times10^{6}(52\rho/\Delta\nu)(\nu_{p}-4155)\times(7.19\times10^{9}-\nu_{p}^{2})^{-2}$ (5)

where *ρ* is the gas density in amagats (defined as ideal gas molecules per unit volume at ambient pressure), *ν_p_* is the pump laser frequency in inverse centimeters (cm^-1^) and *Δν* is the FWHM of the Raman gain profile in megahertz (MHz). It should be noted that Eqation (5) is valid for room temperature (298 K)^13^.

**Supplementary Note 2: Raman laser simulation**

The SRS process is associated with two regimes: the steady-state and transient Raman regime. The latter has a lower Raman gain coefficient than the former being the main obstacle towards high efficiency and pulse energy. The impact of the transient Raman regime is especially dominant when the pump pulse duration is comparable or less than the dephasing time (~0.3 ns) of the H_2_ molecules^14^. A common approach to suppress the transient Raman regime is to reduce the dephasing time by increasing the H_2_ pressure (e.g. 50 bar, as in Ref. [14, 15]). Although this approach can - to some extent - suppress the transient Raman regime, the quantum efficiencies reported are still less than 60% ^14, 15^. Moreover, the requirement of high H_2_ pressure also limits the laser’s usability and stability. Therefore, an alternative way to effectively suppress the transient Raman regime is by increasing the duration of the pump pulses.


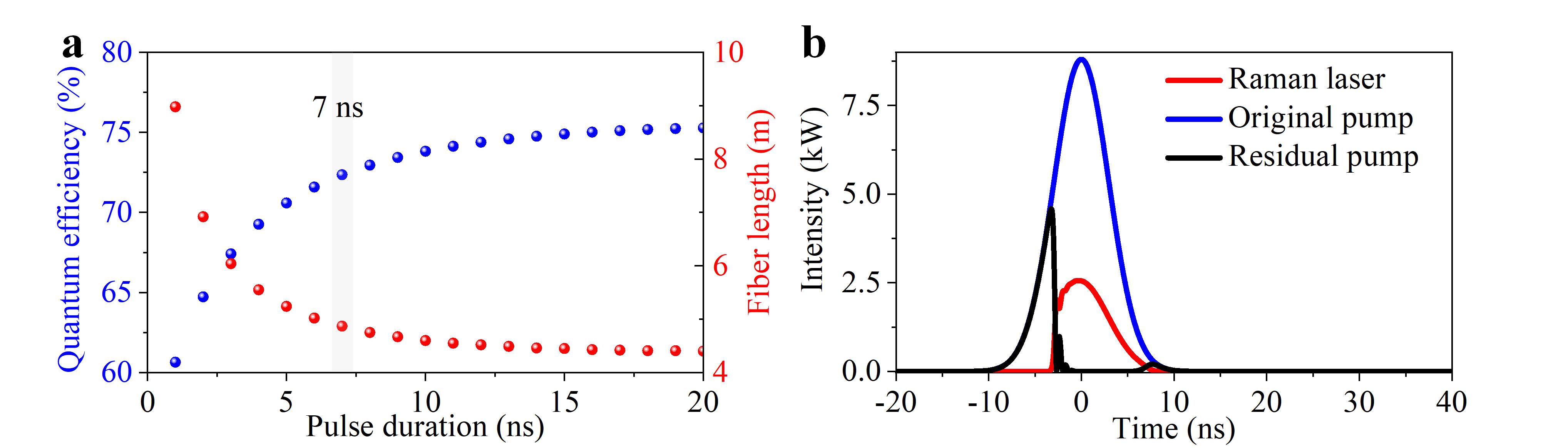


**Supplementary Figure S1:** Simulation of the gas-based excitation source at 4.22 µm. (**a**) Quantum efficiency and optimal ARHCF length at different pulse durations. The pump pulse peak power is set to 11 kW, coupling efficiency inside the ARHCF to 80%, fiber losses to 0.01 dB/m at the pump wavelength of 1533 nm and 0.27 dB/m at Raman laser wavelength of 4.22 µm, mode-field diameter of anti-resonant fiber to 51 µm and H_2_ pressure to 15 bar. (**b**) Pulse profiles of the original pump laser (blue), residual pump (black), and Raman laser (red) simulated under 7 ns pump pulses.

Numerical modeling was performed to investigate the quantum efficiency and the corresponding optimum ARHCF length as a function of the pump pulse duration, as depicted in Supplementary Fig. S1a. It shows that with increasing of the pulse duration, the quantum efficiency first continuously increases and then tends to saturate, indicating the progressive suppression of the transient Raman regime. The use of long pump pulse duration also leads to high pulse energies of the pump and thus the generated beam. However, it should be noted that practically – given the damage threshold being a limitation - the use of long pump pulse duration is associated with low peak power, which also significantly compromises the final laser’s efficiency. Therefore, the pump pulse duration should be optimally chosen in order to achieve efficient suppression of the transient Raman regime, having also sufficiently high peak power. Our modeling indicates that the optimum duration of the pump pulses should be 7 ns, which corresponds to a quantum efficiency of 72.3 %, approaching to the saturated quantum efficiency of 74.2 %. Supplementary Fig. S1b shows the pulse profiles of pump (before HCF), residual pump (after HCF), and Raman laser under 7 ns pump pulse duration. The calculated pulse energy of the Raman laser is up to 16.0 µJ. The Raman laser pulse exhibits a narrower pulse duration (6.1 ns) than the original pump pulse. It is attributed to the high threshold of the SRS effect: for the Gauss pump pulse, only the center part of the pump pulse profile with instant power higher than the SRS threshold can enable the SRS ^14^.Accordingly, the residual pump pulse exhibits two narrow sub-pulses that correspond to the leading and trailing edges of the original pump pulse. The difference between the two pulses is a sign that there is still weak residual transient Raman regime ^14^.

**Supplementary Note 3: Simulation of anti-resonant HCF**

The HCF is designed with a 73 µm air-core region, surrounded by a ring of 7 capillaries with a wall thickness of 1.03 µm and a diameter of 43.6 µm, as shown in the Supplementary Fig. S2a. The simulation was performed using the finite-element method (software COMSOL). To ensure the accuracy of our simulation, the mesh was set with fine size from λ/6 to λ/4 and the boundary conditions were set as perfectly-matched layer to accurately determine the leakage loss. The absorption coefficient of fused silica used in the simulation is from Ref. [16] while the modeling parameters are similar to Ref. [17]. Supplementary Figure S2 shows the optical loss of the fiber as a function of wavelength. The simulation result shows two transmission bands separated by a strong loss peak centered at ~2 µm due to capillary resonance, as depicted by the calculated transmission spectrum in Supplementary Fig. S2b. The calculated losses at the pump wavelength of 1.53 µm and signal wavelength of 4.22 µm are as low as 0.01 dB/m and 0.27 dB/m, respectively. Compared with the pump wavelength, the relatively high loss at signal wavelength is attributed to the high silica loss at MIR.


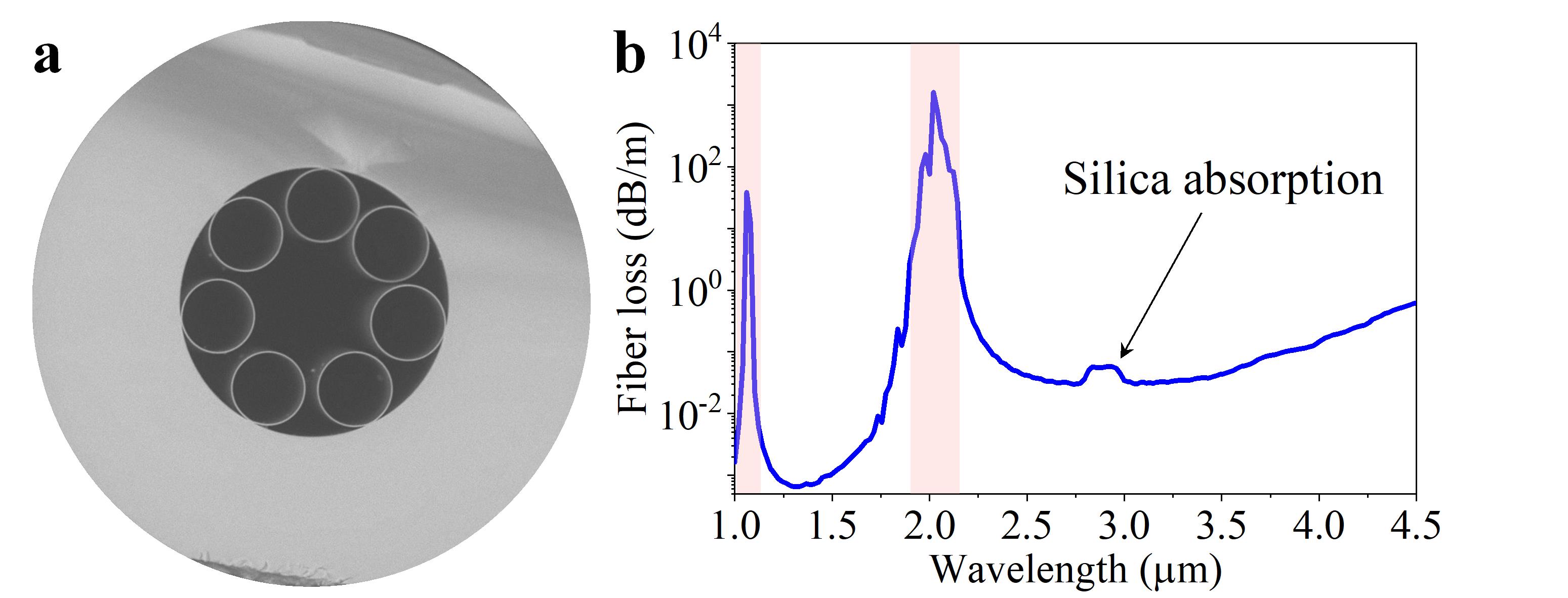


**Supplementary Figure 2S:** ARHCF structure, as well as attenuation spectrum simulation. (**a**) SEM image of the HCF with a magnification of ×400. (**b**) Simulated attenuation spectrum of the HCF. The highlighted shaded regions indicate the resonances of the fiber.

**Supplementary Note 4: Raman laser power evolution as a function of H_2_ pressure**

According to the optimization analysis as described in Supplementary Note 3, the pump laser is designed and developed with 7 ns pulse duration to effectively suppress the transient Raman regime. The ARHCF length was experimentally optimized to 3.95 m, to obtain the highest Raman power of 141 mW, corresponding to a high quantum efficiency of 74% and high pulse energy of 17.6 µJ. Note that this Raman laser power was determined by considering the losses of the long-pass filter, CaF_2_ windows, and the CO_2_ absorption in ambient air. In this case, the measured average output laser power first quickly scales up when the H_2_ pressure exceeds ~5 bar, and then steadily reaches a saturation level. The complete saturation was achieved at ~20 bar, as shown in Supplementary Fig. S3a. The power saturation at this high H_2_ pressure is attributed to the reduced dephasing time, as well as the saturation of the Raman gain at the steady Raman region^13^, as calculated in Supplementary Fig. S3b. The reduced dephasing time of H_2_ combined with the long pump pulses efficiently suppress the transient Raman regime, and thus makes the steady Raman regime dominant. On the other hand, the gain coefficient at the steady Raman regime of H_2_ is almost saturated after 10 bar, consequently saturating the output power.

**
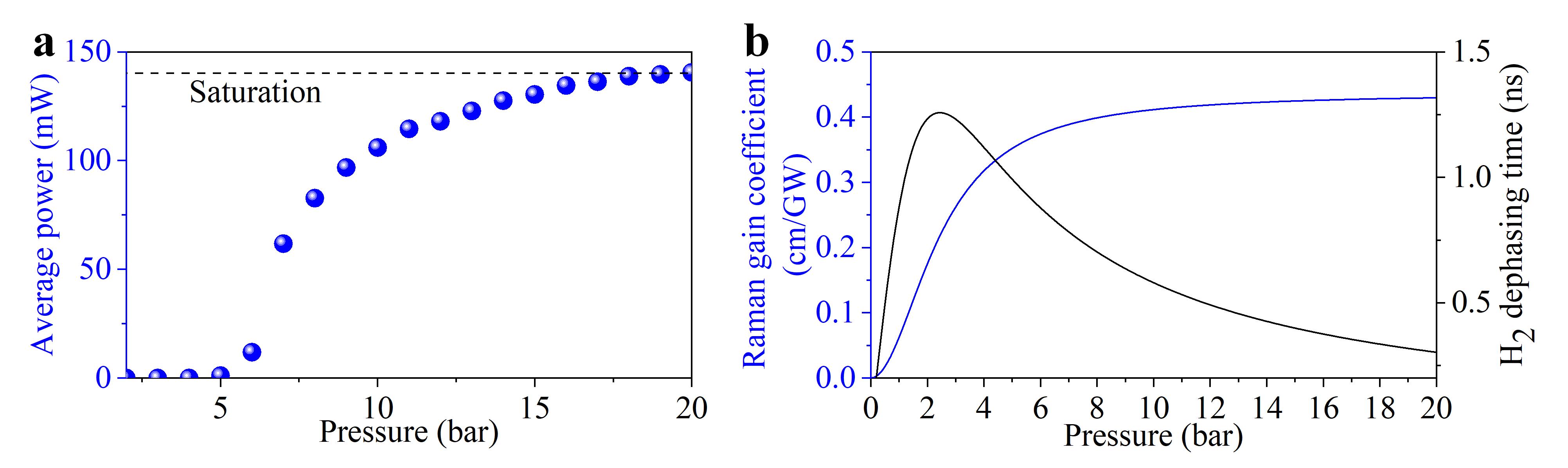
**

**Supplementary Figure 3S.** Power saturation of the gas-based fiber Raman laser. (**a**) Measured average power of as a function of H_2_ pressure. (**b**) Theoretical Raman gain coefficient in the steady-state regime, and dephasing time of H_2_ as a function of pressure.

**Supplementary Note 5: Attenuation measurement of generated MIR laser power in ambient conditions**

The generated MIR laser beam lies on top of the strongest absorption of CO_2_, a key gas component in the atmosphere. Therefore, the laser shows an apparent attenuation when it propagates in air of ~408 ppmv CO_2_ ^18^. In order to investigate the open-path absorption of CO_2_, we measured the attenuation of the Raman laser power as a function of propagation distance, assuming constant CO_2_ concentration, as shown in the Supplementary Fig. S4. During the measurement, the Raman laser beam was collimated by a CaF_2_ lens with 5 cm focus length, and the power is measured using a thermal power meter with a detection diameter of 1 cm, which is much larger than the beam size. By fitting the experimental result with an exponential function, the attenuation coefficient is estimated to be ~ 0.86 m^-1^.

**
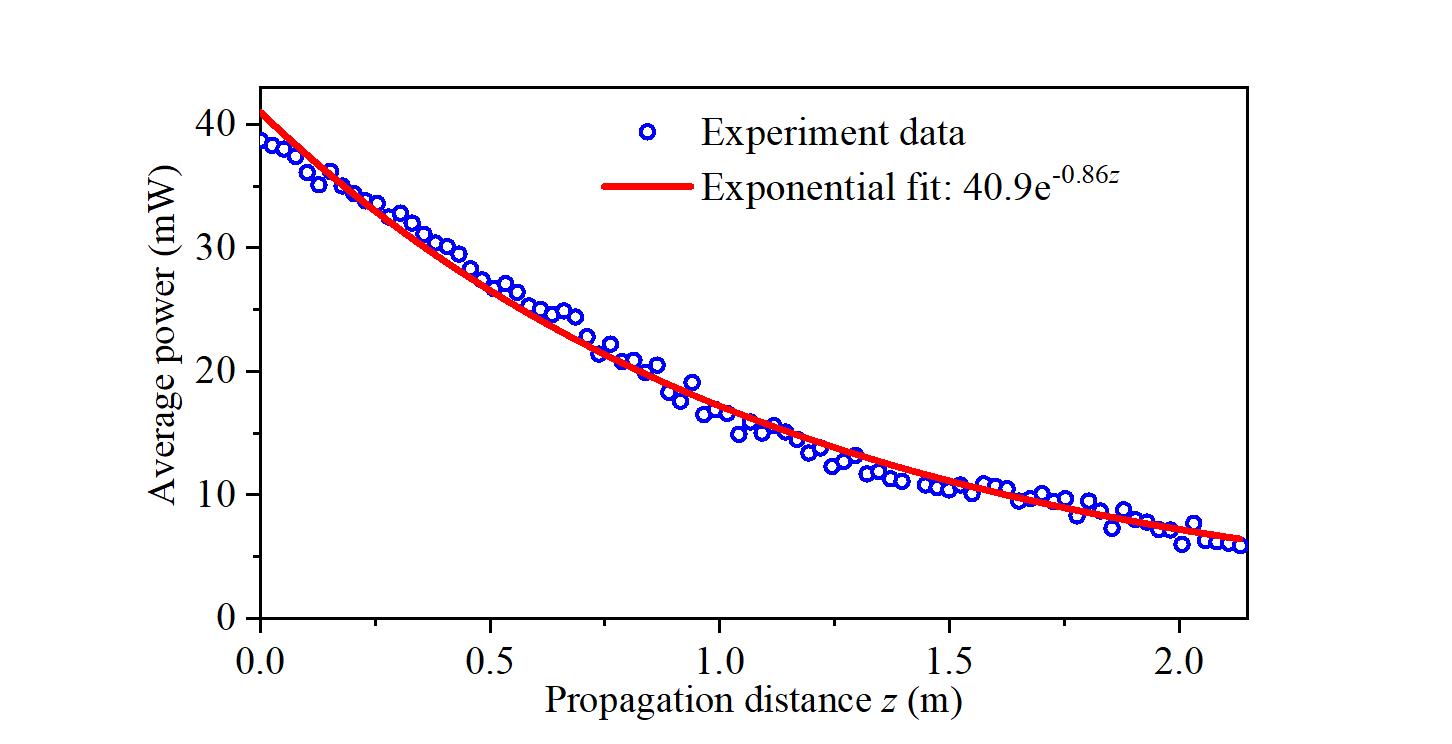
**

**Supplementary Figure 4S.** Open-path direct absorption measurement of CO_2_ in the ambient air. The red curve corresponds to an exponential fit.

**Supplementary Note 6: Impact of CO_2_ concentration on MIR laser power**

The gas absorption induced laser attenuation obeys the Beer-Lambert law:

$P=P_{0}\exp(-\alpha Cz)$ (6)

where *α* is the absorption coefficient, *z* is the propagation distance, and *C* is gas concentration. In our experiment, because the compact PA cell has a short length of only ~10 cm, and CO_2_ concentration is less than 500 ppmv, the absorption loss is therefore negligible. For instance, Supplementary Fig. S5 shows a comparison between the evolution traces of the average output power of the Raman laser and the PA intensity as a function of CO_2_ concentration from 500 ppmv to 1 ppmv. It can be seen that the Raman laser power slightly varies from 18.1 mW to 20.3 mW with high fluctuation, while the PA intensity has a regular and clear evolution trace.

**
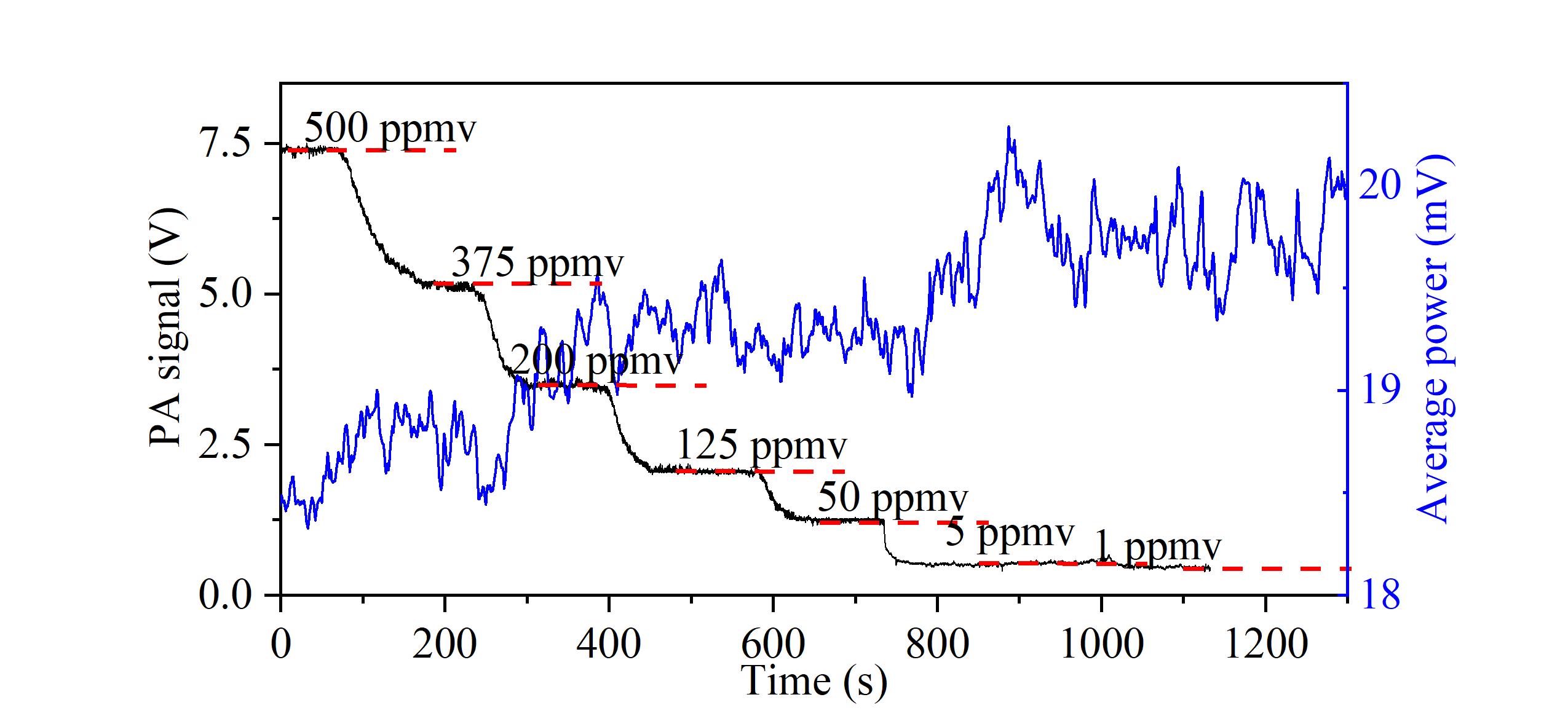
**

**Supplementary Figure 5S.** Evolution traces of PA intensity and Raman laser power as a function of CO_2_ concentration.

**Supplementary Note 7: Pulse instabilities and noise effect**

Since the SRS process is built-up from the random quantum noise^19, 20^, the Raman laser exhibits relatively high pulse-to-pulse fluctuation, as indicated by the comparison of the pump and Raman laser pulse trains in Supplementary Fig. S6a and S6b. The relative standard deviation of pulse energy calculated over 5000 consecutive pulses, is ~ 3 % for the pump laser while ~ 17% for the Raman laser. As a result, the average power of the proposed Raman laser also shows high fluctuations when compared to the pump laser, as shown in Supplementary Fig. S6c. The relative standard deviation of the Raman laser power is calculated to be 1.34%, which is ~6.7 times higher than that of the pump laser. The randomly distributed instabilities of the average power is associated with a drift of the PA signal, consequently resulting in a short optimal integration time of 3.9 s during the calculation of the Allan deviation as shown in Fig. 3a, where the data points beyond the optimal integration time reflecting the effect of the drift of the PA system. The Raman laser instabilities can be interpreted as follows: It is known that the SRS conversion process from 1.53 µm (pump) to 4.22 µm (target) is associated with a large amount of heat-release due to the large quantum defect that transfers 64 % of a pump photon energy into heat (estimated to be ~330 mW in our experiment); the released heat then leads to the increase of the H_2_ temperature inside the ARHCF. When this process is combined with the high pulse-to-pulse fluctuation caused by the quantum noise, the heat energy released from each pump pulse becomes different from each other, which causes the uncertain and relatively slow thermal variation inside the ARHCF. Since the Raman gain of H_2_ is temperature-dependent ^13, 21, 22^, the temperature variation results in the instability of final Raman gain and thus, the output average power.


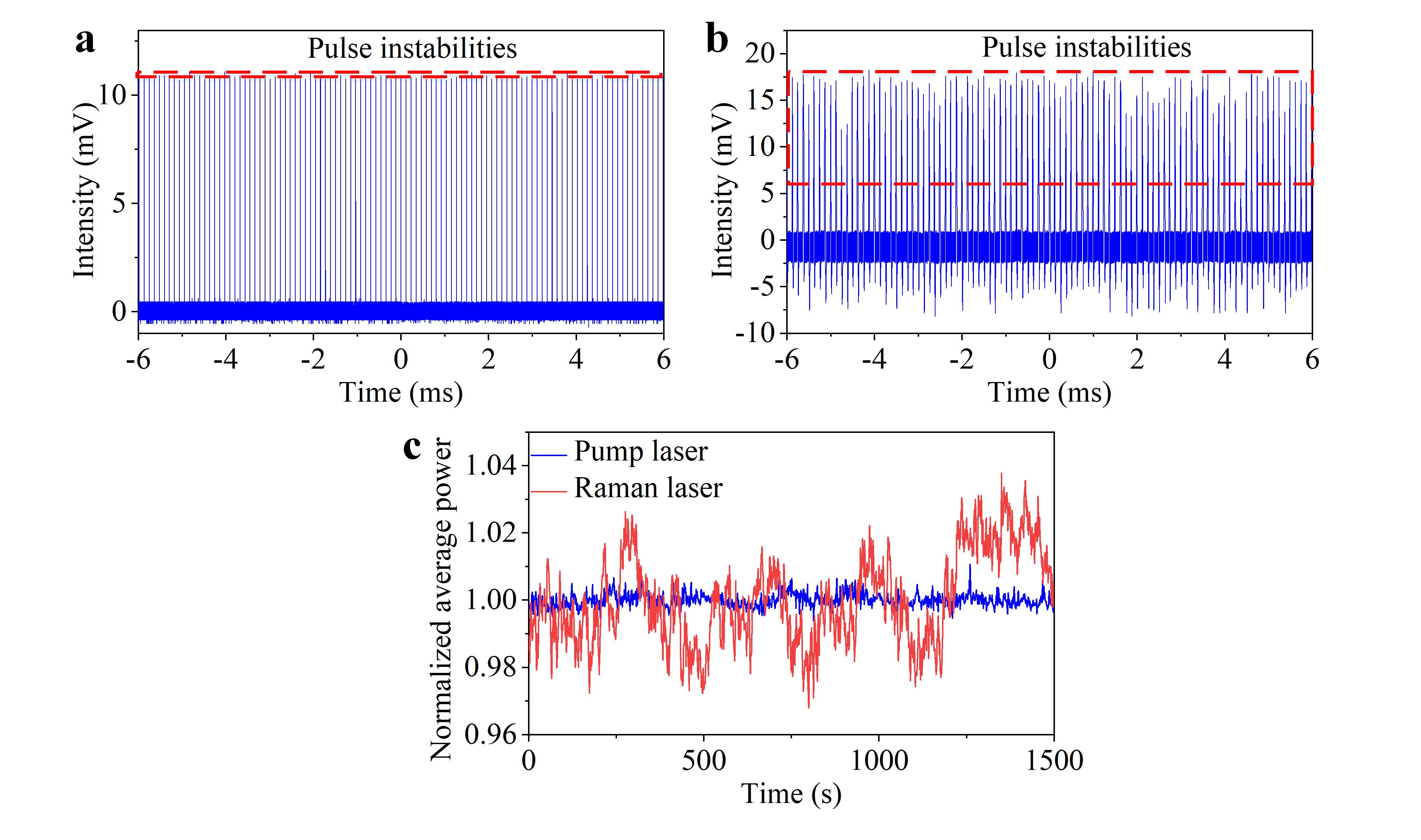


**Supplementary Figure 6S.** Noise characteristics of the Raman laser. (**a**) and (**b**) are measured pulse trains of the pump laser and Raman laser, respectively; (**c**) Stability of the initial pump and the generated MIR laser with respect time. To facilitate direct comparison, their average values are normalized to 1.

**References**

1. Hodgkinson, J. *et al*. Non-dispersive infra-red (NDIR) measurement of carbon dioxide at 4.2 μm in a compact and optically efficient sensor. *Sensor. Actuat. B-Chem.* **186,** 580-588 (2013).

2. Lendl, B., Ritter, W., Harasek, M., Niessner, R. & Haisch, C. Photoacoustic monitoring of CO_2_ in biogas matrix using a quantum cascade laser. Sensors, 2006 IEEE, 338-341 (2006).

3. Spearrin, R. M., Ren, W., Jeffries, J. B. & Hanson, R. K. Multi-band infrared CO_2_ absorption sensor for sensitive temperature and species measurements in high-temperature gases. *Appl. Phys. B-Lasers O.* **116,** 855-865 (2014).

4. Wang, J., Niu, X., Zheng, L., Zheng, C. & Wang, Y. Wireless mid-infrared spectroscopy sensor network for automatic carbon dioxide fertilization in a greenhouse environment. *Sensors* **16,** 1941 (2016).

5. Barritault, P. *et al.* Low power CO_2_ NDIR sensing using a micro-bolometer detector and a micro-hotplate IR-source. *Sensor. Actuat. B-Chem.* **182,** 565-570 (2013).

6. Dong, M., *et al.* Development and measurements of a mid-infrared multi-gas sensor system for CO, CO_2_ and CH_4_ detection. *Sensors* **17,** 2221 (2017).

7. Wolff, M., Germer, M., Groninga, H. G. & Harde, H. Photoacoustic CO_2_ sensor based on a DFB diode laser at 2.7 μm. *Eur. Phy.s J. Spec. Top.* **153,** 409-413 (2008).

8. Galli, I., *et al.* Spectroscopic detection of radiocarbon dioxide at parts-per-quadrillion sensitivity. *Optica* **107**, 385-388 (2016).

9. Koskinen, V., Fonsen, J., Roth, K. & Kauppinen, J. Cantilever enhanced photoacoustic detection of carbon dioxide using a tunable diode laser source. *Appl. Phys. B-Lasers O.* **86**, 451-454 (2007).

10. Borri, S., *et al.* Intracavity quartz-enhanced photoacoustic sensor. *Appl. Phys. Lett.* **104**, 091114 (2014).

11. Lewicki, R., Wysocki, G., Kosterev, A. A., Tittel, F. K. Carbon dioxide and ammonia detection using 2 μm diode laser based quartz-enhanced photoacoustic spectroscopy. *Appl. Phys. B-Lasers O.* **87,** 157-162 (2007).

12. Hilfer G. & Menyuk C. R. Stimulated Raman scattering in the transient limit. *J. Opt. So.c Am. B* **7,** 739-749 (1990).

13. Bischel, W. K. & Dyer, M. J. Wavelength dependence of the absolute Raman gain coefficient for the Q(1) transition in H_2_. *J. Opt. Soc. Am. B* **3,** 677-682 (1986).

14. Gladyshev, A. V. *et al*. Efficient 4.42 μm Raman laser based on hollow-core silica fiber. *arXiv:*1801.01729 [physics.optics] (2017).

15. Astapovich, M. S. et al. Watt-level nanosecond 4.42- µm Raman laser based on silica fiber. *IEEE Photonic. Tech. L.* **31,** 78-81 (2019).

16. Yang, S. T. *et al.* Comparing the use of mid-infrared versus far-infrared lasers for mitigating damage growth on fused silica. *Appl. Opt.* **49,** 2606-2616 (2010).

17. Habib, M. S., Antonio-Lopez, J. E., Markos, C., Schülzgen, A. & Amezcua-Correa, R. Single-mode, low loss hollow-core anti-resonant fiber designs. *Opt. Express* **27,** 3824-3836 (2019).

18. https://www.climate.gov/news-features/understanding-climate/climate-change-atmospheric-carbon-dioxide.

19. Raymer, M. G. & Walmsley, I. A. III the quantum coherence properties of stimulated Raman scattering. In: Wolf E (ed). *Prog. Optics* **28,** Elsevier, 181-270 (1990).

20. Landahl, E., Baiocchi, D. & Thompson, J. R. A simple analytic model for noise shaping by an optical fiber Raman generator. *Opt. commun.* **150,** 339-347 (1998).

21. Hanna, D., Pointer, D. & Pratt, D. Stimulated Raman scattering of picosecond light pulses in hydrogen, deuterium, and methane. *IEEE J. Quantum. Elect.* **22,** 332-336 (1986).

22. Bischel, W. K. & Dyer M. J. Temperature dependence of the Raman linewidth and line shift for the Q(1) and Q(0) transitions in normal and para-H_2_. *Phys. Rev. A* **33,** 3113-3123 (1986).
